# Dyslipidemia: Genetics, lipoprotein lipase and HindIII polymorphism

**DOI:** 10.12688/f1000research.12938.2

**Published:** 2018-10-01

**Authors:** Marcos Palacio Rojas, Carem Prieto, Valmore Bermúdez, Carlos Garicano, Trina Núñez Nava, María Sofía Martínez, Juan Salazar, Edward Rojas, Arturo Pérez, Paulo Marca Vicuña, Natalia González Martínez, Santiago Maldonado Parra, Kyle Hoedebecke, Rosanna D’Addosio, Clímaco Cano, Joselyn Rojas

**Affiliations:** 1 Endocrine and Metabolic Diseases Research Center, School of Medicine, University of Zulia, Maracaibo, Venezuela; 2 Hospital Básico de Paute, Public Health Ministry, Paute, Ecuador; 3 Grupo de Investigación Altos Estudios de Frontera (ALEF), Universidad Simón Bolívar, Cúcuta, Colombia; 4 Department of Medicine, Rutgers University, Newark, NJ, 07103, USA; 5 WONCA Polaris - USA, Bangkok, 10500, Thailand; 6 Yongsan Health Clinic, Seoul, 96205, South Korea; 7 Department of Public Health, School of Medicine, University of Zulia, Maracaibo, Venezuela; 8 Division of Pulmonary and Critical Care Medicine, Brigham and Women’s Hospital, Harvard Medical School, Boston, MA, 02115, USA

**Keywords:** Dyslipidemia, Polymorphisms, HindIII, Lipoprotein Lipase, coronary artery disease

## Abstract

The direct link between lipid metabolism alterations and the increase of cardiovascular risk are well documented. Dyslipidemias, including isolated high LDL-c or mixed dyslipidemia, such as those seen in diabetes (hypertriglyceridemia, high LDL-c or low HDL-c), correlate with a significant risk of cardiovascular and cerebrovascular disease worldwide.  This review analyzes the current knowledge concerning the genetic basis of lipid metabolism alterations, emphasizing lipoprotein lipase gene mutations and the HindIII polymorphism, which are associated with decreased levels of triglycerides and LDL-c, as well as higher levels of HDL-c. These patterns would be associated with decreased global morbidity and mortality, providing protection against cardiovascular and cerebrovascular diseases.

## Dyslipidemia: The current status

The relationship between dyslipidemia and atherosclerosis continues to be an area of active research, since the prevalence of atherosclerosis and associated cardiovascular complications continue to increase in the industrialized world ^1^. Cardiovascular disease (CVD) constitutes the greatest cause of morbidity and mortality globally with a high incidence in countries of all economic categories ^2^. Evidence supporting a causal relationship between lipid profile abnormalities and the risk of coronary artery disease (CAD) is overwhelming, confirming that hypercholesterolemia is an independent risk factor for CVD ^3– 5^. In addition, hypertriglyceridemia and mixed dyslipidemias have been associated with the aggregation of metabolic risk factors, like hypertension (HTN) ^6^ and obesity ^7^.

Dyslipidemias are a group of metabolic derangements characterized by any or a combination of the following: elevated low density lipoprotein (LDL-c) (>130md/dL), elevated total cholesterol (>200 mg/dL), elevated TG (>150mg/dL), or low high density lipoprotein (HDL-c) (<40mg/dL in men and <50mg/dL in women) ^8^.

The worldwide prevalence of dyslipidemia varies between different individuals, depending on race, age, socio-economic and cultural factors, lifestyle and genetics. This prevalence has increased significantly in growing cities with economic growth ^9^. The factors are undoubtedly related to high calorie intake described by other Western nations ^10^; while lower prevalence has been reported for these pathologies in Canada and South Korea where 45% and 44.1% of their respective populations present evidence of dyslipidemia ^11, 12^. The difference of prevalence in these nations is indisputably tied to lifestyle with various publications highlighting diets rich in fiber and low in fats and refined sugars ^13, 14^.

In Brazil, de Souza *et al*. 2003. ^15^ reported the most frequent dyslipidemias in their 1039 sample population were isolated low HDL-C (18.3%), hypertriglyceridemia (17.1%), and isolated hypercholesterolemia (4.2%). These results differ from those reported in the CARMELA study from Mexico between 2003 and 2005 ^16^, where the incidence of dyslipidemia in 1722 person sample size reported 16.4% with hypercholesterolemia and 32.5% with hypertriglyceridemia - slightly higher than those reported in Brazil. The differences could be related to the food preferences within Mexico, which include greater amounts of fat, simple sugars, and processed ingredients ^17^. It must also be noted that these studies are over 10 years old as no more recent publications in Latin America were found. With the continued rise in dyslipidemias and obesity, these numbers do not necessarily reflect the current reality of these pathologies in the studied populations.

In Venezuela, the CARMELA study evaluated the prevalence of these lipid metabolism disorders in the city of Barquisimeto. The researchers found 59.6% of the 1824 subjects to suffer from dyslipidemia in 2003 ^18^. A decade later, Linares *et al*. ^19^ published a study of 1892 sample size from the city of Maracaibo, Venezuela showing a prevalence of 84.8% - the highest figure reported to date. The difference of prevalence is related to both the ten year gap between the studies as well as the regional differences between the sample populations. Even though these two studies occurred in Venezuela, the two sites have different cultures, climate, and completely different nutritional food preferences. The diet of the latter is traditionally high in calories, protein, and carbohydrates with an elevated alcohol intake ^20^ while only 40% of the former ever ate fast food or other foods outside the home ^21^.

In the majority of the studies of prevalences there is a clear tendency to evaluate younger populations, (22–24 years of age) as in the study by Barja *et al*. between 2009–2011 ^23^ where 2900 school-age children were evaluated with the average age of 11.42±0.97 years old. Of this sample, 9.4% had isolated hypertrigliceridemia, 7.6% with low HDL-C, 4.9% with isolated hypercholesterolemia, 6.24% with atherogenic dyslipidemia, and 3.9% with mixed dyslipidemia. The existence of hyperlipidemia increases the potential for deposits on the tunica intima and the formation of arterial plaques. A correct early diagnosis of dyslipidemia allows for timely preventive intervention resulting in a reduction cardiovascular disease and facilitates the patient’s clinical treatment ^25^.

## Dyslipidemia genetics

The association between family history of dyslipidemia and the risk of CVD is supported by a large body of evidence ^18– 22^. Additionally, the great advancement in DNA analysis techniques has aided research surrounding CVD and related genetics and epigenetics. Understanding gene mutations or polymorphisms involved in the synthesis, transport, and metabolism of lipoproteins allows recognition of potential therapeutic targets and alternative treatments through identification of new molecules ^1, 3, 20^.

Dyslipidemia is one of the most well characterized cardiovascular risk factors ^19, 20^. This not only depends on diet, but also on the synthesis and metabolism of lipoproteins conditioned by gene expression. Given the importance and the great diversity of proteins that participate in lipid metabolism, one might expect that a single defect in any step of gene expression would affect the quantity or quality of the product and potentially predispose to dyslipidemias and CVD ^19^.

One genetic abnormalities associated with low HDL-c and increased CVD risk is the *Taq IB* polymorphism located in chromosome 16q21. This gene alters cholesteryl ester protein transferase (CEPT), which decreases HDL-c concentration ^23^. Some deletions, inversions, and substitutions of the *APO AI-IV*, *CII*, and *CIII* genes are also associated with both premature CVD and low HDL-c ^24, 25^. Total deficiency of lecithin cholesterol acyl transferase (LCAT) can be seen after transition of C→T in codon 147 of exon 4 (W147R), G→A in codon 293 of exon 6 (M293I), as well as partial deficiencies of LCAT due to transition of C→T. Additionally, the substitution of threonine for isoleucine in codon 123 (T123I) causes decreased HDL-c and higher cholesterol in the intima of arterial vessels ^26, 27^.

Below, some of the genetic alterations associated with low levels of HDL-c and a higher risk of CVD are highlighted:

• *CETP:* This mediates the exchange of lipids between lipoproteins. With high levels of *CETP*, HDL are transitioned into triglycerides (TGs), becoming the substrate for hepatic lipase where TGs are hydrolyzed. Apoproteína (Apo) A-1 is degraded in tubular renal cells and diminishes the amount of HDL-C - increasing the atherogenic potential. The polymorphism rs1801706 (c.*84G>A) of the *CETP* gene is associated with CAD ^27^.
• *Familial hypoalphalipoproteinemia and HDL-C deficiency:* Approximately 50% of the HDL-C alterations are explained by polygenic defects in various chromosomal loci that control apolipoprotein expression (A-I, A-II, C-II, C-III y A-IV). Multiple genetic variations such as deletions, inversions, and substitutions of gene coding for apolipoproteins are associated with severe CAD ^28, 29^.
• *LCAT:* This liver-synthesized enzyme circulates in plasma forming complexes with HDL and participating in the inverse transport of cholesterol. LCAT deficiencies cause an accumulation of free cholesterol in tissues. One of the most recent described gene alterations is the P-274-S polymorphism that afects biogenesis of HDL-C ^30^ and favors development of CVD.
• *ABCA*1: This protein mediates the transport of cholesterol and phospholipids from the cells to LDL. The C-69-T polymorphism of the gene codifies this protein and alterations can result in lower levels of HDL-C with higher levels of TG in obese children ^31^. Additionally, the rs2515602, rs2275542, rs1800976, and rs4149313 polymorphisms are associated with obesity and can negatively affect one’s lipid profile base on one study performed on 535 Chinese patients ^32^.
• *FTO:* This includes a group of 45 genes related to obesity that were grouped together during phylogenetic analysis ^33^ and perform an important function in the regulation of food ingestion. *FTO* mutations are associated with obesity, metabolic syndrome and CAD ^34^. The literature does not specify which lipid metabolism genes affect *FTO*, but the rs9939609 polymorphism is associated with low HDL-C levels.

The following are some genetic alterations associated with hypercholesterolemia and hypertriglyceridemia, including their relationship with increased cardiovascular risk:

• *LDLR* gene - LDL-C receptor and familial hypercholesterolemia (FH): LDL-C is a macromolecular complex that transports cholesterol and cholesteryl esters from the liver to other peripheral tissues, where cholesterol is introduced to the cells through LDL receptors (LDLR) ^36^. FH, an autosomal dominant condition caused by mutations on the *LDLR* gene, is one of the best characterized genetic defects ^37, 38^. Mutations on this lipoprotein or one of the proteins involved in its metabolism in duce hypercholesterolemia and elevated LDL-C - both factors predisposing to the premature CAD development.
• *APO B-100* gene – ligand of LDL-C receptor and Familial Apolipoprotein B dysfunction (hypercholesterolemia type B). *ApoB-100* is the principle apolipoprotein in LDL and the ligand of LDLR. The autosomal dominant dislipidemia know as Familial *APO B-100* Dysfunction (FDB) and is due to mutations in *ApoB-100* ^39^. Recently two new mutations on this gene have been described - p.Arg1164Thr and p.Gln4494. These variants have problems with binding to LDLR ^40^ where carriers of this mutation bear a greater cardiovascular risk.
• APO E gene – Apolipoprotein E and hyperlipoproteinemia or hyperlipidemia type III. Apolipoprotein E (ApoE) is a principal component of chylomicrons (CMs), very low density lipoprotein (VLDL) and some HDL-C. Its main function is to serve as the ligand for hepatic receptors for the remnants of the aforementioned lipoproteins and regulation of VLDL production. Alterations in this apoprotein cause hyperlipoproteinemia or Type III Hyperlipidemia (HLP III) where the plasma levels of cholesterol and triglycerides are increased ^26^. The polymorphisms in ApoE are associated with variations in plasma cholesterol levels where individuals with this allele mutation exhibit cholesterol levels 10% greater than average ^41^.
• *LPA Gene:* This is formed by a nucleus rich in cholesterol esters, phospholipids, and an ApoB-100 that contains a union site for LDL receptors, but still contains an ApoA molecule. Lp(a) consists of one of the most important cardiovascular risk factors and proves of greater significance when correlated with elevated levels of LDL-C - observed commonly in patients under 60 years old with CVA ^42^. Polymorphisms have been found consisting of variable repetitions of module 4 where the number of repetitions is inversely proportional to the plasma levels of Lp(a) ^43^. Recent work has encountered differences in the distribution of the diverse alleles of ApoA among patients with atherosclerosis and the isoforms of low weight molecular B, S1, and S2. These are found most frequently in carriers of coronary insufficiency who also show elevated levels of Lp(a) ^44^. This suggests that the short alleles of ApoA contribute to atherogenesis, increasing the plasma concentration of Lp(a).
• HL gene - hepatic lipase and phenotype of combined familial hyperlipidemia. Combined familial hyperlipidemia is a genetic lipid disorder that accounts for 10–20% of premature CAD worldwide. Affected individuals’ exhibit hypercholesterolemia and/or hypertriglyceridemia and elevated concentrations of APO B, with low values of HDL-c. These are collectively called iatrogenic lipoproteinemia phenotype. There have been demonstrations of alterations in common genetic loci between families of both combined familial hyperlipidemia phenotype and atherogenic lipoproteinemia phenotype. (ALP). Such loci include genes of superoxide manganese dismutase, transport proteins of cholesteryl esters/lecithin, cholesterol acyl transferase and AI-CIII-AIV, as well as a great variety of studies relating polymorphisms in the promoter region of the LH gene (C-480T and C-514T polymorphisms) with lowering on plasma levels of HDL-C ^45, 46^.
• *ApoCIII: Apoprotein CIII*: This Apo inhibits the activity of LPL. It is a component of the lipoprotein remnants that possess elevated TG levels. The loss of function of this Apo has been associated with low TG levels and a reduction of coronary artery calcification ^47^. The polymorphism C3175>G localized in region 3` presents in less than 5% of the population in the UK. Those who present with this polymorphism have elevated TGs and deficient LPL - augmenting the probability of developing CVA. Currently the pharmaceutical companies Isis and Ionis have developed an antisense oligonucleotide the blocks ARNm fora ApoCIII. This is currently in phase 2 of clinical trials and preliminary studies show a significant decline of TGs in patients with ApoCIII mutations ^48^.
• LPL gene - lipoprotein lipase, Apo CII and familial dyslipidemia type O or familial chylomicronemia. Any mutations on the LPL gene, which results in a partial deficiency of the enzyme, will cause an increase in TG concentration. This is the basis of familial chylomicronemia, familial dyslipidemia type I or familial hypertriglyceridemia; These are monogenic diseases with autosomal recessive inheritance, consisting with pure hypertriglyceridemia, TG values of 300 to 800 mg/dl, cholesterol <240 mg/dl, increases in VLDL and CMs, and lowering of LDL-C and HDL-C. To date, some LPL variants have been characterized because of amino acids substitutions in different positions (D9N, N291S, substitutions of glycine for glutamine on codon 188 and serine for a termination signal on codon 4) ^47^. The enzymatic activity of LPL is also lowered by mutations of the ApoCII gene, an essential activator of LPL. Specifically, the mutation R72T of the ApoCII gene causes severe hypertriglyceridemia and recurrent pancreatitis ^49^.

This information justifies the use of genetic markers for early diagnosis and cardiovascular risk assessment, especially in children and adolescents, in order to adopt early nutritional or pharmacologic interventions with the aim to mitigate atherosclerotic artery disease.

## Lipoprotein lipase

**Figure 1.**
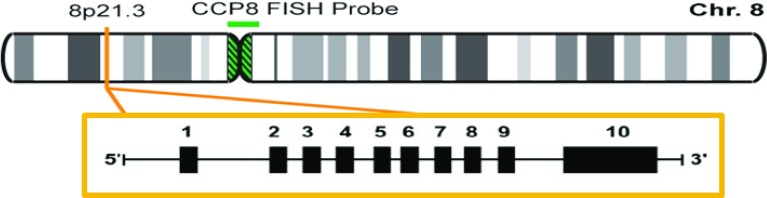
Chromosomal origin of the LPL gene. The authors confirm that this is an original image and has not been re-used or adapted from another source.

The *LPL* gene is located on the short arm of chromosome 8, on the region 21.3 (8p21.3). It is formed of 10 exons and 9 introns ( Figure 1), and the gene codifies a protein of 475 amino acids ^53, 54^.

*LPL* is a multifunctional glycoprotein enzyme that plays an important role on lipid metabolism. After being secreted, it adheres to the luminal surface of endothelial cells where it hydrolyzes TG in circulating lipoproteins. This constitutes the limiting step on lipoprotein elimination, such as CMs from exogenous sources, and those endogenous sources, like VLDL, in circulation ^55, 56^.

In this way, *LPL* affects serum levels of TG, generating lipoprotein remnants that are processed by hepatic lipase. Recently, it has been demonstrated that *LPL* serves as a ligand for the protein related to the LDLR and influences hepatic secretion and VLDL and LDL-c capture ^57^. Additionally, *LPL* has been linked to the retention of LDL-c by the sub-endothelial matrix and arterial wall, increasing LDL and VLDL conversion into more atherogenic forms ^58^. Genetic modifications can affect *LPL* activity, which results in changes in lipid metabolism. Examples are slow hydrolysis of CMs and VLDL-c, longer LDL-c half-life, and decreased production of HDL ^59, 60^.

Around 100 mutations have been described on the *LPL* gene. The most frequent are Asp9sn, Gly188Glu and Asn291Ser. The mutations in the homozygous form are associated with hyperlipoproteinemia type I (familial chylomicronemia). Heterozygous mutations have a significant incidence in the general population (3–7%) and leads to up to a 50% decreased activity of *LPL*, causing an increase in TG and a decrease in HDL-c. All these lipid profile patterns increase the risk of CVD ^61^.

## *LPL* gene polymorphisms

Genetic studies have revealed around 100 mutations and polymorphisms in simple nucleotides on the *LPL* gene, some are protective, which others are deleterious:

*1.*  *Ser447x (rs328) polymorphism* is located in exon 9, where cytosine is substituted by guanine on position 1959. This polymorphism leads to the suppression of both final amino acids, serine and glycine on position 447 of the protein that codifies a *LPL* protein prematurely truncated, which has increased lipolytic activity and increased levels of post-heparin LPL activity in X447 carriers. This is associated with the variant Ser447X, with low levels of TG, small increases of HLD-C levels, and a moderate CVD risk reduction ^62^. These results differ from those of Emamian *et al.* ^61^ who studied 271 obese individuals and reported elevated TGs in carriers of this polymorphism. In studies of postprandial lipids, it has been reported that the aforementioned carriers present with elevated blood glucose and TGs than non-carriers ^62^. Thes reports clearly indicate that the benefit of this mutation are limited in patients of normal weight under the evaluated conditions.
*2.*  *PvuII (rs285) polymorphism,* located on intron 6, is located 1.57 kb from the Splicing Acceptor (SA) site. This polymorphism is the product of a change of cytosine for thymine. The region containing the PvuII site is similar to the splice location. This suggests that a change at C497-T may interfere with the correct splicing of mRNA. Even though the physiological role associated with this polymorphism is not completely clear, it has been associated with high TG and low HDL-C levels ^63^. A meta analysis revealed that this polymorphism reduces the risk of suffering from an MI ^64^ and, therefore, appears to have a protective effect against CVA.
*3.*  *HindIII (rs320) polymorphism* is one of the most common polymorphisms of *LPL* gene (see below).

## *HindIII* (rs320) polymorphism

**Figure 2.**
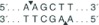
Recognition sequence of *HindIII* enzyme. The authors confirm that this is an original image and has not been re-used or adapted from another source.

**Figure 3.**
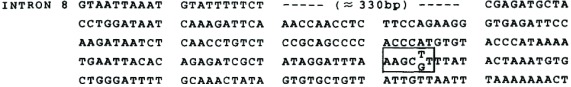
Intron 8, restriction site of *HindIII* (AAGC TT > AAGC GT). The authors confirm that this is an original image and has not been re-used or adapted from another source.

*HindIII* is a transition of intronic bases of thymine (T) to guanine (G) on position 495 of intron 8 of the *LPL* gene, which eliminates the restriction site for the *HindIII* enzyme ( Figure 2 and Figure 3). Sequential analysis has determined that this HINDIII recognition site corresponds with the binding consensus sequence for the transcription factors Sp1, GATA, C/EBP, and TBP. The first three are implicated in the regulation of the gene transcription involved in lipid metabolism ^65– 67^. The transcription factor TBP (the binding protein for the TATA box) initiates the formation of the preinitiation complex that permits gene transcription for part of RNA polymerase II ^68^. Mobility shift electrophoresis has shown that human vascular smooth muscle cells and COS-1 cell carriers of the G/G allelele demonstrated reduced TBP binding affinity ^69^. THis indicates less LPL expression in polymorphism carriers, conferring functionality of said SNP.

*HindIII* is one the most frequent polymorphisms found in various studies, which show that the homozygous genotype T/T (H ^+^/H ^+^) represents from 45.1 to 56.4% of Iranian and south Indian populations, respectively most frequent, followed by the heterozygous T/G with 35.8–36.6% and homozygous G/G (H ^-^/H ^-^), with 6.93–19% ^64, 65^. Similar results have been reported in Europe ^66, 67^ and Brazil ^68^.

**Figure 4.**
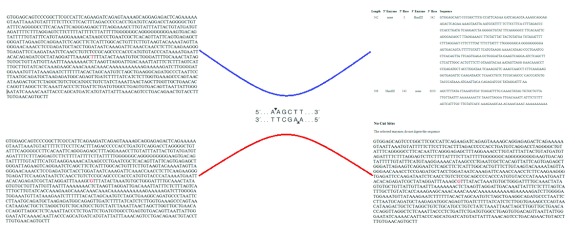
Enzymatic restriction sites in *HindIII* ^69^. The authors confirm that this is an original image and has not been re-used or adapted from another source.

The allele H+ (presence of thymine “T” or restriction site of *HindIII* enzyme) results in a cut on the base pair sequence in two bands of 217pb and 139pb. This is associated with a decrease in the activity of *LPL* in comparison with the allele H- (presence of “G” or absence of the enzymatic restriction site or presence of *HindIII* polymorphism). With 137pb, in which there is no cut in the *LPL* gene intron 8 sequence, maintaining a unique sequence of 356pb ( Figure 4) ^69^, leading to both alterations in lipidic metabolism and cardiovascular risk profile modifications in these populations.

Some studies have demonstrated that the common allele (T or H+) is associated with lower levels of HDL-c in contrast with the uncommon allele (G or H-) ^70, 71^. In addition, those individuals with H+/H+ genotype had a higher concentration of serum levels of TG when compared with homozygous genotype H-/H- ^66, 67, 70, 72^. Similarly, there have been reports of high serum levels of LDL-c ^71^ and a higher global cardiovascular risk in patients who carry the common allele (T or H+), see Table 1. Some studies had reported a significant drop in the *LPL* activity among carriers of the uncommon G allele when compared with the more common allele T ^57^.

**Table 1.** Lipid disorders according *LPL* gene allele ^66, 67, 70– 72^.

Serum lipid levels	Triglycerides	LDL	HDL
**Common allele (H+)**	High	High	Low
**Uncommon allele (H-)**	Low	Low	High

*LPL* expressed by macrophages and other cells contained in the vascular walls is involved in the early atherogenic process and is associated with increased atherosclerosis. Overexpression of *LPL* is also associated with insulin resistance and HTN by increased sodium retention, inflammation, vascular remodeling, sympathetic nervous system activation, oxidative stress and vasoconstriction ^73– 75^.

On the other hand, HTN (mostly systolic) has been shown to be associated with the polymorphism *HindIII* in the Mexican population in studies by Muñoz-Barrios *et al*. ^76^. Similarly, the homozygous genotype for the common allele (H+) was associated with a higher risk of myocardial infarction in patients older than 90 years old in contrast with carriers of the uncommon allele (H-), associated with a lower prevalence of cardiovascular complications ^77^. Clear associations were found between genotypes of *LPL HindIII* with HTN (H+/H+ with an OR: 2.13; 95% CI: 0.93-4.8) ^72^ and smoking ^58^. In a more recent study, it was established that the presence of homozygous genotype for the common allele (H+/H+) of the *LPL* gene is a risk factor for a first episode of myocardial infarction ^65^. Conversely, studies by Imeni *et al*. ^78^ in an Iranian population, showed no statistically significant associations between CAD and genotypic distributions of *HindIII* polymorphism.

Recent studies have shown increased risk of stroke among those with LPL gene variations, particularly in the *HindIII* gene ^79^. He *et al*. reported a lower risk of stroke among patients with *HindIII* polymorphisms with allele G (G vs T; OR=0.78, CI95%=0.70-0.87, p<0.001). This pattern was observed in patients with ischemic stroke (G vs T. OR=0.84, CI95%=074-0.95, p=0.005) and hemorrhagic stroke (G vs. T; OR=0.60, CI95%=0.48-0.74, p<0.001) ^80^.

In other studies, Imeni *et al.* ^86^ evaluated the relationship between CAD risk and the distribution of HindIII polymorphism genotypes and found no statistical significant differences between healthy Irani individuals and those with CAD history. Ahmadi *et al.* ^87^ also showed no significant association between the gene and CAD in the 115 Swiss subjects evaluated. These findings are in contrary to the expected improved cardio-cerebral function expected and leave this line of research open for future investigations.

This polymorphism has not only been associated with HA, rather also with insulin resistance. This is best demonstrated with a study of 110 Asian females with gestational diabetes who were found to have a reduced resistance to insulin than carriers of this rare allele ^88^.

From a neurologic point of view, there is scant data associating homozygous common genotype (H+/H+) with the development of Alzheimer’s disease of late appearance. This is founded on the *LPL* function in regulation cognitive function, mediated by cholesterol and Vitamin E transport to neuronal cells on the hippocampus and other brain areas ^64^.

These investigations suggest that the presence of the HindIII polymorphism exerts a positive influence in lipid metabolism in patients with normal BMI. Future studies should focus in more detail on the protective function of this genetic factor in the general population.

## Conclusions

Dyslipidemias are independent risk factors for atherosclerotic artery disease. High TC, TAG and LDL-C, as well as decreased serum HDL-C, are frequently associated with low physical activity and poor eating habits, but there is a large number of mutations and single nucleotide polymorphism related to a specific protein dysfunction within major lipoprotein metabolism pathways like CETP, ApoA, LCAT, LDL receptor, Apo B-100 and LPL.

In this regard, the *LPL* gene *HindIII* polymorphism (rare allele H-) poses a protective function through its role in producing an improved lipid profile (low TG and LDL-c and high HDL-c). On the other hand, the presence of common allele (T or H+) is associated with pro-atherogenic dyslipidemias and raised cardiovascular risk. The uncommon allele (G or H-) with an absence of restriction *HindIII* enzyme exhibits a lower prevalence of at least 20% according to the current available literature.

There are no studies in Venezuela that allows us to know the true prevalence of the HindIII polymorphism, nor to corroborate the association with changes in the lipid profile or an increased risk for cardiovascular diseases, so we suggest performing a national populational genetic study in search for this lipidic disorders with the aim to has a better understanding of the cardiovascular risk factors in Latin America.

## Data availability

All data underlying the results are available as part of the article and no additional source data are required.
