# Supplementary material for: Dyslipidemia: Genetics, lipoprotein lipase and HindIII polymorphism
Source: F1000Res. 2018 Oct 1;6:2073. Originally published 2017 Nov 30. [Version 2] doi: 10.12688/f1000research.12938.2 (PMC6171722; doi:10.12688/f1000research.12938.2)
Supplement: Peer review history

# Peer review: Referee response for version 2

**DOI:** 10.5256/f1000research.17787.r38893

**Published:** 2018-10-01

**Authors:** Carlos Aguilar-Salinas

**Affiliations:** 1 Instituto Nacional de Ciencias Médicas y Nutrición, Unidad de Investigación de Enfermedades Metabólicas, México City, Mexico

No additional comments.

I have read this submission. I believe that I have an appropriate level of expertise to confirm that it is of an acceptable scientific standard.

# Peer review: Referee response for version 1

**DOI:** 10.5256/f1000research.14029.r31173

**Published:** 2018-03-05

**Authors:** Carlos Aguilar-Salinas

**Affiliations:** 1 Instituto Nacional de Ciencias Médicas y Nutrición, Unidad de Investigación de Enfermedades Metabólicas, México City, Mexico

Authors of the paper "Dyslipidemia: Genetics, lipoprotein lipase and HindIII polymorphism” summarizes several papers published by Latin American researchers about lipid disorders. The document highlight the need for more studies about genetics of dyslipidemia in this region.  

     

 The main limitations of the study are:

- The paper covers a large number of topics. As a result, information is presented without a critical analyses. Since the  HindIII polymorphism is the main issue under review, a large proportion of the review could be summarized and the HindIII polymorphism data extended. For example, the genes involved in familial hypoalphalipoproteinemia are enlisted partially (i.e. ABCA1 was not mentioned). It is not clear the reason to devote several paragrapjs for FH genes when they are not related with the main topic of this review.
- Reasons for the large differences in the prevalence of the lipid disorders between Latin-American surveys could be critically discussed.
- The style of the manuscript could be upgraded. The flow of the information should be improved.
- It is kindly suggested to consider a redesign of the structure of the document.

I have read this submission. I believe that I have an appropriate level of expertise to confirm that it is of an acceptable scientific standard, however I have significant reservations, as outlined above.

# Peer review: Referee response for version 1

**DOI:** 10.5256/f1000research.14029.r30011

**Published:** 2018-01-19

**Authors:** David J. Galton

**Affiliations:** 1 Wolfson Institute of Preventive Medicine, The Royal London and St. Bartholomew's Hospitals, London, UK

This review deals with the following genes involved in lipid metabolism: CETP, LCAT, LDL receptor, apoE, Lp(a), hepatic lipase and lipoprotein lipase. However it misses out details of the apoC3 gene which is the only one in which a specific therapy (volanesorsen) has been developed. A review of this can be found in Galton (2017) ^1^.

 The authors then go on to deal with the Hind 111 polymorphism of Lipoprotein lipase. A common *Hind*III polymorphism in intron 8 (T/G) of the LPL gene has been found to be associated with altered plasma TG and HDL-cholesterol, and CAD risk in several studies, but they do not comment on its functional significance.

 It is known that certain intronic sequence contain regulatory elements that are important for transcription and translational regulation of a gene. A recent study (Chen et al. (2008) ^2^) showed that this Hind 111 polymorphism affects the binding site of a transcription factor that regulates the transcription of LPL gene. Electrophoretic mobility shift assays revealed that the *Hind*III site binds to a transcription factor and that the mutant allele has lower binding affinity than the wild type allele. Transcription assays containing the entire intron 8 sequence along with full-length human LPL promoter were carried out in COS-1 and human vascular smooth muscle cells. The mutant allele was associated with significantly decreased luciferase expression level compared to the wild type allele in both the muscle (3.394 ± 0.022 vs. 4.184 ± 0.028; P=4.7 × 10 ^−6^) and COS-1 (11.603 ± 0.409 vs. 14.373 ± 1.096; P<0.0001) cells. This study demonstrates for the first time that the polymorphic *Hind*III site in the LPL gene is functional because it affects the binding of a transcription factor and it also has an impact on LPL expression.

I have read this submission. I believe that I have an appropriate level of expertise to confirm that it is of an acceptable scientific standard.

## References

[1] GaltonDJ: Clarifying complex inheritance: apolipoprotein C3 and atherosclerosis. *Curr Opin Lipidol*.2017;28(4) : 10.1097/MOL.0000000000000425 308-312 10.1097/MOL.0000000000000425 28441154

[2] ChenQRazzaghiHDemirciFYKambohMI: Functional significance of lipoprotein lipase HindIII polymorphism associated with the risk of coronary artery disease. *Atherosclerosis*.2008;200(1) : 10.1016/j.atherosclerosis.2007.12.011 102-8 10.1016/j.atherosclerosis.2007.12.011 18242618PMC2603421
